# Association between frailty and chronic constipation and chronic diarrhea among American older adults: National Health and Nutrition Examination Survey

**DOI:** 10.1186/s12877-023-04438-4

**Published:** 2023-11-15

**Authors:** Xuna Liu, Yiwen Wang, Lin Shen, Yating Sun, Beibei Zeng, Boxu Zhu, Fei Dai

**Affiliations:** 1 Department of Gastroenterology, The Second Affiliated Hospital of Xi’an Jiaotong University, Xi’an, 710004 Shaanxi China; 2 Xi’an International Medical Center Hospital Affiliated To Northwest University, Xi’an, 710119 China

**Keywords:** Frailty index, Chronic constipation, Chronic diarrhea, Stool frequency, NHANES

## Abstract

**Background:**

This study was to explore the relationship between chronic constipation, chronic diarrhea, and frailty in older Americans.

**Methods:**

This cross-sectional study selected a total of 4241 community-dwelling individuals aged 60 years and older from the 2005–2010 National Health and Nutrition Examination Survey. Frailty was measured using a 49-item frailty index, and a frailty index > 0.21 was defined as a frail status. Chronic constipation and chronic diarrhea were defined as the “usual or most common type of stool” by the Bristol Stool Form Scale (BSFS) Types 1 and 2 and BSFS Types 6 and 7, respectively. Weighted logistic regression analysis was used to examine the relationship between gut health and frailty status. Restricted cubic spline (RCS) curves were built to assess the association between frailty index and stool frequency.

**Results:**

Frailty status was associated with higher odds of constipation in an unadjusted model; however, after further adjusting for confounding variables, the relationship between frailty status and constipation was not statistically significant. We discovered a positive correlation between the frailty status and diarrhea after adjustment for all variables. The frailty index showed a U-shaped relationship with stool frequency, and the frailty index was the smallest at a frequency of 10 stools/week.

**Conclusion:**

Negative associations were observed between frailty status and chronic constipation and diarrhea among older adults. Older adults who have a bowel movement frequency of about 10 times per week are the least frail. Future studies are warranted to confirm the causal relationship in this association.

**Supplementary Information:**

The online version contains supplementary material available at 10.1186/s12877-023-04438-4.

## Introduction

Frailty is defined as a decreased function of multiple physiological systems and increased vulnerability to stressors, resulting in elevated mortality, as well as a heightened probability of hospitalization, falls, and receipt of long-term care [1]. The rapidly growing elderly population brings with it an increase in the number of frail elderly people, so frailty is the most prominent manifestation of population aging [2]. Frailty is still preventable, although prevention of frailty in the period before death may be of little importance [3]. Therefore, it is vital to prevent and slow the progression of frailty. Numerous studies have been devoted to the development of tools for the objective assessment of frailty. Fried’s phenotype model and the frailty index model are the two main models. According to Fried’s phenotype model, older adults who fulfilled three or more of the five physical criteria were diagnosed as frailty [4]. The frailty index model formulated by Lockwood was grounded in the accumulation of age-related deficits, including chronic diseases, psychosocial factors, cognitive deficits, and other signs and symptoms of aging [5].

Bowel symptoms, including constipation and diarrhea, are common in the elderly population. The prevalence of chronic constipation and diarrhea is estimated to be as high as 17% and 20% in the general population, with a higher prevalence in the elderly [6, 7]. The prevalence of self-reported constipation in the community is 20 to 40% in elderly individuals [8]. Chronic diarrhea is defined as loose or watery stools greater than 25% of the time [9], while chronic constipation is characterized by fewer than three defecations per week and hard, dry or lumpy stools [10]. The occurrence of constipation and diarrhea is influenced by various factors, such as age, sex, lifestyle, body condition, and diet [11]. Constipation and diarrhea can hurt the quality of life and increase healthcare costs [12, 13]. Despite this, constipation and diarrhea in older adults have been neglected and are often undertreated or poorly treated in routine clinical practice.

Bowel symptoms may exacerbate frailty through different pathways such as the absorption of nutrients, intestinal motility, and gut microbiota [14, 15]. Although previous studies have shown an association between constipation and frailty [16–18], the samples were mostly from Asian populations, dietary intake as a potential influence on this relationship was not adequately studied, and most studies used Fried's phenotype to define frailty. In addition, no research has been done on the relationship between diarrhea and frailty. Therefore, we used data from the National Health and Nutrition Examination Surveys (NHANES) 2005–2010 to examine the relationship between frailty and chronic constipation and diarrhea.

## Materials and methods

### Population

**Fig. 1:**
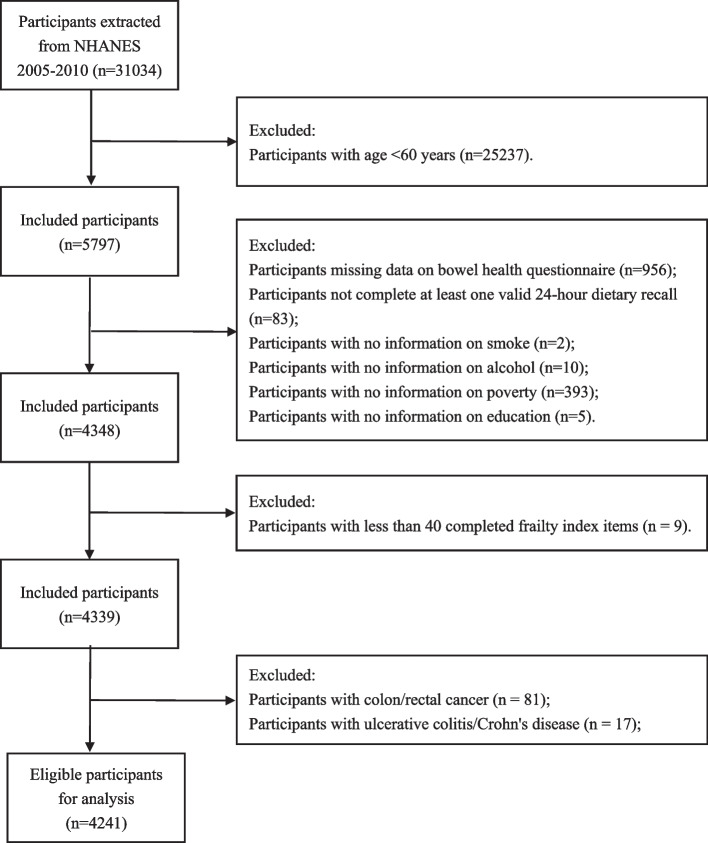
Flowchart of the sample selection from NHANES 2005–2010

NHANES is a publicly accessible research study project performed by the National Center for Health Statistics at the Centers for Disease Control (Atlanta, GA, USA) that includes a nationally representative sample of noninstitutionalized subjects. Participants were chosen using a hierarchical, multistage, probabilistic clustering design and then analyzed using complex sampling weighting methods [19]. Written informed consent was provided by all participants, and parental consent was obtained if participants were under 18 years of age. NHANES was approved by the National Center for Health Statistics’ Ethics Review Board. All procedures were conducted under relevant guidelines and regulations (https://www.cdc.gov/nchs/data_access/restrictions.htm). The study population for this analysis included subjects from the 2005–2010 National Health and Nutrition Examination Survey (NHANES) cycle. We chose these three cycles because data on bowel health questionnaires are not available in the recent cycles. A total of 31,034 participants were extracted from the NHANES database. After excluding participants aged < 60 years (*n* = 25,237), missing data on the bowel health questionnaire (*n* = 956), not completing at least one valid 24-h dietary recall (*n* = 83), and insufficient baseline information (property (*n* = 393), education (*n* = 5), smoking (*n* = 2), alcohol use (*n* = 10)), 4348 participants were included. Further, participants with less than 80% (< 40 items) of the characteristics of the included frailty index were rejected (*n* = 9). Finally, participants with colorectal cancer (*n* = 81), and Crohn’s disease/ulcerative colitis (*n* = 17) were eliminated, and 4241 participants were eligible for analysis (Fig. 1).

### Frailty assessment

The frailty index devised by Hakeem and his coworkers was used to evaluate frailty in our study [20]. The Frailty Index assessment system includes seven sections with 49 items on cognition, dependence, depression, comorbidities, hospital utilization and access to care, physical performance and anthropometry, and laboratory values [20]. To make it possible to combine continuous variables with categorical variables, a score between 0 and 1 was distributed depending on the severity of the deficit. The frailty index is equal to the score of deficits obtained by participants divided by the total score of potential deficits (Supplementary Table 1 in the Additional file 1). According to the frailty index cutoff value of 0.21, more than or equal to 0.21 is frailty and less than 0.21 is non-frailty [21].

### Bowel health questionnaire

The NHANES 2005–2010 bowel health questionnaire was applied to analyze bowel symptoms in participants who self-reported their bowel habits. We evaluated stool consistency using Bristol Stool Form Scale (BSFS) cards (color picture cards with pictures and written descriptors of the seven stool types) and the following written questions: “Please look at this card and tell me the number that corresponds with your usual or most common stool type.” Individuals with chronic constipation are considered to have the usual or most common type of stool identified as BSFS type 1 (separate hard lumps like nuts) or BSFS type 2 (like sausage, but with lumps) [22]. Individuals with chronic diarrhea are considered to have the usual or most common type of stool identified as BSFS type 6 (fluffy pieces with ragged edges, a mushy stool) or BSFS type 7 (watery, no solid pieces) [9]. The stool frequency assessment question was "How many bowel movements do you usually have per week?”.

### Co-variables

Certain covariates formerly shown or hypothesized to be related to chronic diarrhea and/or constipation and frailty were evaluated as covariates in our study, including sociodemographic variables, behavioral risk factors, and dietary intake. The sociodemographic factors were age, measured in years; sex; race, categorized into five groups (non-Hispanic white races, non-Hispanic black, Hispanic, Mexican American, and other race—including multiracial); education, divided into three levels (less than high school, high school or General Education Diploma (GED), and greater than high school); marital status, classified into three groups (married/living with a partner, never married, separated/divorced/widowed); and poverty to income ratio, used as a continuous variable to represent income. Behavioral risk factors comprised smoking and alcohol consumption. Smoking status was divided into three groups (never smokers “smoked less than 100 cigarettes in life”, former smokers “smoked more than 100 cigarettes in life and do not smoke now”, and current smokers “smoked more than 100 cigarettes in life and smoke now”). Alcohol users were divided into “yes/no” groups. Overall dietary quality was assessed using the 2015 version of the Healthy Eating Index (HEI). HEI—2015, scored according to the Dietary Guidelines for Americans (DGA), consists of 13 food blocks or nutrients that correspond to their recommended intakes. There are nine adequacy components in the HEI (including total fruits, whole fruits, total vegetables, greens and beans, whole grains, dairy, total protein foods, seafood and plain proteins, and fatty acids) and four moderation components (including refined grains, sodium, added sugars, and saturated fats). For each component, there is a maximum score of 5 or 10, with a total score of 100, with higher scores indicating better overall diet quality (Supplementary Table 2 in the Additional file 1). Finally, we also collected data on the total daily energy intake of the subjects.

### Statistical analysis

For the study, appropriate weights were used (i.e., 1/3 * 2-year mobile examination sampling weights) to account for the complex sampling design of NHANES. Descriptive statistics were performed stratified by frailty or not. A t-test was used to compare differences between groups for continuous data with a mean (standard error) (S.E). A chi-square test was used to determine differences between groups based on categorical data expressed as number (n) and percentage (%). The correlation between independent variables was analyzed using Spearman correlation analysis. It is generally accepted that correlations are classified as strong, moderate, weak, and no correlation by the correlation coefficient (ρ) with three cutoff values of 0.8, 0.5, and 0.3. Weighted logistic regression analysis was conducted to investigate the association between frailty index and chronic constipation and diarrhea. The crude model was unadjusted. In Model 1, age, sex, race education, marital status, and poverty-income ratio factors were adjusted. In Model 2, smoking and alcohol use were additionally adjusted. In Model 3, HEI and total energy intake were further adjusted. Restricted cubic spline (RCS) regression adjusted for all confounding variables was used to examine the linear/nonlinear relationship between frailty index and stool frequency. If the association was nonlinear, the inflection point of stool frequency on the frailty index was sought, i.e., the value at which change was observed in the curve. All statistical analyses were performed using R version 4.2.1 (R Foundation for Statistical Computing, Vienna, Austria; http://www.r-project.org), and statistical significance was determined by a two-sided *P* value < 0.05.

## Results

### Prevalence in the whole population

The study included 4241 individuals representing 402.5 million non-institutionalized residents in the United States. The prevalence of frailty among U.S. seniors over 60 was 24.49% [95% confidence interval (CI) 21.65–27.33], chronic constipation was 6.66% (95% CI 5.69–7.62), and chronic diarrhea was 8.01% (95% CI 6.44–9.58). Among them, the prevalence of chronic constipation was lower in older men than in older women (9.74% (95% CI 8.55–10.93) vs. 2.97% (95% CI 2.30–3.64), *p* < 0.0001), as was chronic diarrhea (9.11% (95% CI 7.53–10.69) vs. 6.70% (95% CI 5.26–8.13), *p* = 0.02).

### Baseline characteristics

**Fig. 2:**
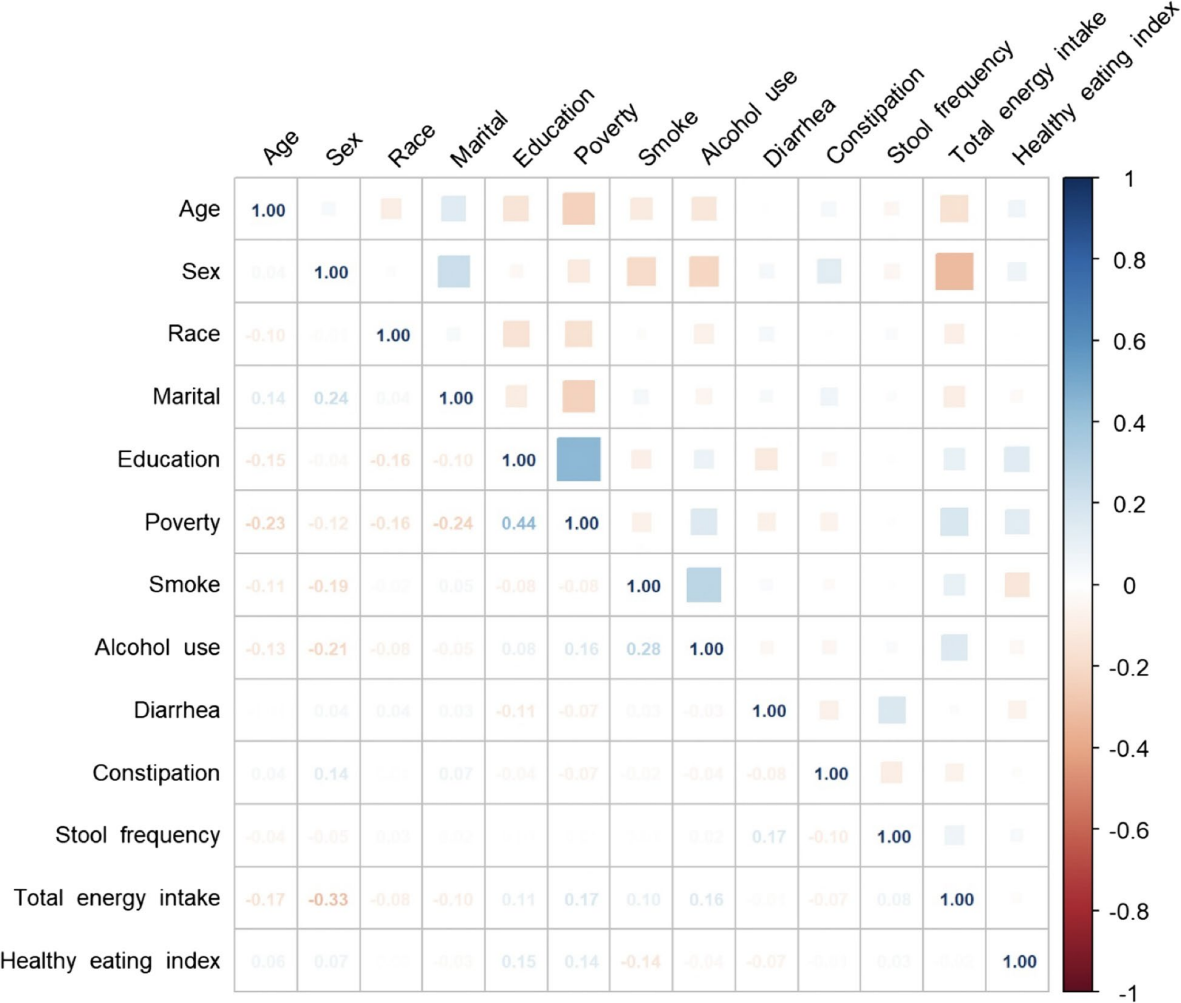
Spearman correlation among independent variables

The baseline characteristics of the research participants are presented in Table 1. The average age of the study population was 69.91 ± 0.16 years, and 48.97% were female. Frail participants were more likely to be older (72.34 ± 0.28 vs. 69.12 ± 0.17, *p* < 0.0001) and female (26.68% vs. 21.66%, *p* = 0.001) and had a lower income-to-poverty ratio (2.30 ± 0.06 vs. 3.20 ± 0.05, *p* < 0.0001), total energy intake (1675.68 ± 24.73 vs. 1848.49 ± 21.42, *p* < 0.0001) and HEI score than non-frail participants (51.88 ± 0.62 vs. 55.38 ± 0.36, *p* < 0.0001). Significant differences in race, education, marital status, and alcohol exposure (*p* < 0.05) were also observed. Smokers were more likely to be frail than never-smokers (28.84% vs. 25.19% vs. 22.82%, *p* = 0.05), but the difference was not statistically significant. Frailty was more prevalent in subjects with constipation (35.47% vs. 23.71%, *p* < 0.001) and diarrhea (34.56% vs. 23.61%, *p* < 0.001). In the correlation analysis in Fig. 2, there was no correlation between the independent variables except between education and wealth (ρ = 0.44, *p* < 0.05) and total energy intake and sex (ρ = -0.33, *p* < 0.05).

**Table 1:** Demographic, socioeconomic, and bowel health characteristics of study participants stratified by frailty status

	Total (*n* = 4241)	Non-frailty (*n* = 3047)	Frailty (*n* = 1194)	*P* Value
Age, Mean (S.E)	69.91 (0.16)	69.12 (0.17)	72.34 (0.28)	< 0.0001
Sex n (%)				0.001
Female	2077 (48.97)	1443 (73.14)	634 (26.86)	
Male	2164 (51.03)	1604 (78.34)	560 (21.66)	
Race n (%)				0.001
Non-Hispanic White	2482 (58.52)	1829 (76.94)	653 (23.06)	
Non-Hispanic Black	816 (19.24)	552 (66.09)	264 (33.91)	
Hispanic	275 (6.48)	196 (73.21)	79 (26.79)	
Mexican American	560 (13.2)	394 (69.08)	166 (30.92)	
Other Race	108 (2.55)	76 (72.42)	32 (27.58)	
Marital n (%)				< 0.0001
Married/Living with a partner	2553 (60.2)	1958 (80.08)	595 (19.92)	
Never married	153 (3.61)	108 (72.08)	45 (27.92)	
Separated/Divorced/Widowed	1535 (36.19)	981 (66.72)	554 (33.28)	
Education n (%)				< 0.0001
< High school	1401 (33.03)	872 (62.55)	529 (37.45)	
High school	1065 (25.11)	759 (71.98)	306 (28.02)	
College	1775 (41.85)	1416 (83.55)	359 (16.45)	
Poverty, Mean (S.E)	2.98 (0.05)	3.20 (0.05)	2.30 (0.06)	< 0.0001
Smoke n (%)				0.05
Never	1970 (46.45)	1458 (77.18)	512 (22.82)	
Former	1751 (41.29)	1243 (74.81)	508 (25.19)	
Now	520 (12.26)	346 (71.16)	174 (28.84)	
Alcohol use n (%)				0.01
No	729 (17.19)	480 (69.12)	249 (30.88)	
Yes	3512 (82.81)	2567 (76.71)	945 (23.29)	
Diarrhea n (%)				< 0.001
No	3839 (90.52)	2796 (76.39)	1043 (23.61)	
Yes	402 (9.48)	251 (65.44)	151 (34.56)	
Constipation n (%)				< 0.001
No	3950 (93.14)	2873 (76.29)	1077 (23.71)	
Yes	291 (6.86)	174 (64.53)	117 (35.47)	
Stool frequency, Mean (S.E)	9.02 (0.08)	9.04 (0.09)	8.97 (0.17)	0.74
Healthy eating index, Mean (S.E)	54.52 (0.37)	55.38 (0.36)	51.88 (0.62)	< 0.0001
Total energy intake, Mean (S.E)	1806.17 (20.04)	1848.49 (21.42)	1675.68 (24.73)	< 0.0001

### Association between frailty and constipation and diarrhea

The frailty index was higher in constipated subjects than in non-constipated subjects (0.18 ± 0.01 vs. 0.16 ± 0.00, *p* < 0.001). In patients with diarrhea, the frailty index was also higher (0.19 ± 0.01 vs. 0.16 ± 0.00, *p* < 0.0001).

As shown in Table 2, the weighted logistic regression analysis found a relationship between frailty status and constipation as well as diarrhea. On the one hand, in the crude model, a positive association between constipation and frailty status was found (OR: 1.65, 95% CI: 1.19–2.28). Age, sex, race education, marital status, and poverty-income ratio were adjusted to yield similar results. After further adjusting for smoking and alcohol use, frailty status was associated with a lower likelihood of constipation. Finally, after adjusting for HEI and total energy intake, the relationship between frailty index and constipation was not statistically significant (OR: 1.45, 95% CI: 0.99–2.11, *P* = 0.05). On the other hand, we observed a positive correlation between the frailty status and diarrhea (OR: 1.82, 95% CI: 1.44–2.29). After adjustment for all covariates, a statistically significant positive relationship between frailty status and diarrhea remained (OR: 1.39, 95% CI: 1.06–1.82).

**Table 2:** Weighted logistic regression analysis models showing the associations between constipation and diarrhea and frailty

	Crude model		Model 1		Model 2		Model 3	
	OR (95%CI)	*P* value	OR (95%CI)	*P* value	OR (95%CI)	*P* value	OR (95%CI)	*P* value
BSFS								
Normal	ref		ref		ref		ref	
Constipation	1.65 (1.19,2.28)	0.003	1.48 (1.04,2.09)	0.03	1.49 (1.03,2.16)	0.04	1.45 (0.99,2.11)	0.05
Diarrhea	1.82 (1.44,2.29)	< 0.0001	1.45 (1.12,1.88)	0.01	1.41 (1.08,1.85)	0.01	1.39 (1.06,1.82)	0.02

Crude model: unadjusted model

Model 1: Adjusted for the age, sex, race, education, marital status, and poverty-income ratio;

Model 2: Additionally adjusted for smoking and alcohol use

Model 3: Additionally adjusted for HEI and total energy intake

### Association between frailty and stool frequency

**Fig. 3:**
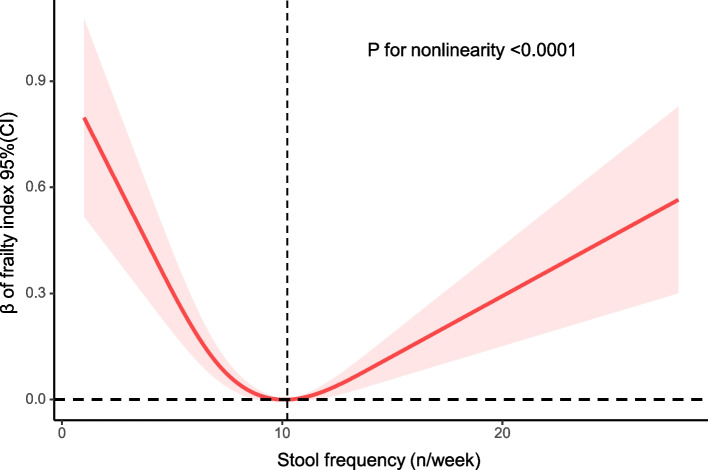
Analysis of restricted cubic spline regression. *Model adjusted for age, sex, race, HEI, total energy intake, education, marital status, povertyincome ratio, smoking, and alcohol use

In a restricted cubic spline plot (RCS), stool frequency was related to the frailty index as shown by a U-shaped curve. (*P* for nonlinearity < 0.0001, Fig. 3). As defecation times increased, β of the frailty index decreased significantly; β of the frailty index was lowest when defecation times reached 10.23 times/week, and then the curve showed an upward trend.

## Discussion

This study demonstrates that chronic constipation and diarrhea were associated with frailty in a nationally representative sample of older Americans. After adjusting for a variety of confounders, we found a positive association between frailty status and diarrhea, and after adjusting for important covariates, the significant association between frailty status and constipation also persisted. In addition, our study displayed a U-shaped relationship between frailty index and stool frequency and found that maintaining a bowel frequency of approximately 10 bowel movements/week minimizes the frailty index.

Previous studies have shown that frailty is associated with chronic constipation in community-dwelling older adults [16–18], which supports our outcomes. Elderly people who suffer from chronic constipation experience pain as well as complications such as hemorrhoids, fecal incontinence, fecal impaction, intestinal perforation, intestinal torsion, rectal prolapse, and excessive perineal or insufficient perineal descent [23], thus affecting the physical health of the elderly and aggravating their frailty [24]. As mentioned in the literature review, elderly individuals with chronic constipation have a higher incidence of depression [25], and depression is an important evaluation aspect of frailty, so constipation may further aggravate frailty in elderly individuals through combination with depression. In addition, chronic constipation can contribute to frailty by causing malnutrition in older adults, and a systematic review of the literature based on longitudinal data showed that constipation is a risk factor for malnutrition in older adults [26]. Therefore, we perceive constipation as a risk factor for frailty.

Irritable bowel syndrome, inflammatory bowel disease, microscopic colitis, and other intestinal disorders may be associated with chronic diarrhea. Several studies have found reduced alpha diversity and dysbiosis of intestinal bacterial flora in patients with chronic diarrhea [27, 28], and this phenomenon has been found in frail patients. Thus, chronic diarrhea may be involved in the development of frailty by altering the intestinal flora in elderly individuals. Patients with chronic diarrhea are in a low-grade inflammatory state with elevated circulating levels of multiple inflammatory factors including IL-6, IL-8, IL-10, IL-1β, TNF-α, etc. [29]. Inflammation can be involved in catabolism associated with adipose tissue and skeletal muscle, which leads to impaired nutrition, muscle weakness, and weight loss, all characteristics of frailty [30]. Unfortunately, our study did not examine the intestinal flora and serum inflammatory factors of the subjects.

The number of bowel movements reflects bowel function and is relevant to a number of diseases. In our study, older adults who had a bowel movement frequency of 10 times per week had the smallest frailty index, which means they were less frail. High or low bowel frequency may affect the quality of life and psychological status of older adults, and high stool frequency has been independently shown to be associated with poorer quality of life among colon cancer survivors [31]. Japanese studies have indicated that lower bowel frequency was associated with higher glycated hemoglobin and with higher risk of CVD death [32, 33]. As this is the first study on frailty and defecation frequency in older adults, the exact mechanism still needs further research in the future. Consistent with earlier studies, we also discovered that total energy intake and healthy eating index were smaller in frail older adults than in non-frail older adults [34, 35]. Inadequate intake of nutrients can lead to the development of many diseases, which can lead to frailty; therefore, nutrition is critical to the health of the elderly [36]. First, older adults may actively choose to avoid certain diets that they consider inappropriate to prevent diarrhea or constipation [37], which leads to malnutrition in older adults. Second, older people prefer processed soft foods, which are rich in sugar and fat but low in vitamins, protein, and minerals [38]. Inadequate micronutrient intake can leave older adults with malnutrition, mitochondrial dysfunction, increased oxidative stress, and low and sterile inflammation, ultimately increasing the risk of frailty [39].

In our study, a high prevalence of frailty in the elderly (24.98% overall) and greater age of frail patients, with a significantly higher prevalence in women (27.48%) than in men in the sex distribution, has been widely reported [2, 17]. The prevalence of frailty varies by race, with the highest prevalence among non-Hispanic blacks and the lowest prevalence among non-Hispanic whites, which may be related to the level of education, social status, and economic level of different ethnic groups [40]. Higher levels of education and income are associated with lower rates of frailty, which may be due to better access to medical resources as well as the stock of medical knowledge. Another risk factor in our study was living alone, which may have increased the risk of depressive symptoms and thus frailty [41]. Alcohol mediates impaired muscle mass and function through multiple mechanisms including reduced protein synthesis, inflammation and oxidative stress, altered myogenic gene expression, bioenergetic transfer, and impaired regenerative capacity [42], which may contribute to frailty in the elderly.

There are some limitations in our study. Primarily, since this study is a cross-sectional study, we cannot conclude a causal relationship between frailty and chronic constipation and diarrhea. In addition, the bowel condition in this study was collected through a questionnaire, which may be affected by recall bias. Moreover, although we adjusted for potential confounders affecting the experimental results, there were still some confounders such as drug administration that were not considered. Despite these limitations, to our knowledge, this is the first study to assess the relationship between chronic diarrhea and frailty index, providing important insights into the connection between constipation, diarrhea, and frailty, and ultimately providing a framework for future prevention and interventions to reduce the risk of frailty by improving gut health.

## Conclusions

In conclusion, we found that frail older adults were more likely to suffer from chronic constipation and diarrhea. A bowel movement frequency of about 10 times per week had the lowest frailty index. It is important to maintain good gut health in older adults, and it is necessary to include gut health in routine health assessments and surveys of older adults.

### Supplementary Information

**Additional file 1: Supplementary Table 1.** The variables in the 49-item frailty index and their respective scoring criteria. **Supplementary Table 2.** Healthy Eating Index -2015 components and scoring standards.

## Data Availability

The datasets generated and/or analyzed during the current study are available in the NHANES database, https://wwwn.cdc.gov/nchs/nhanes/.

## Abbreviations

NHANES: National Health and Nutrition Examination Survey
BMI: Body mass index
HEI: Healthy Eating Index
