# Supplementary material for: Association between frailty and chronic constipation and chronic diarrhea among American older adults: National Health and Nutrition Examination Survey
Source: BMC Geriatr. 2023 Nov 15;23:745. doi: 10.1186/s12877-023-04438-4 (PMC10647084; doi:10.1186/s12877-023-04438-4)
Supplement: Supplementary file 1 — Additional file 1: Supplementary Table 1. The variables in the 49-item frailty index and their respective scoring criteria. Supplementary Table 2. Healthy Eating Index -2015 components and scoring standards. [file 12877_2023_4438_MOESM1_ESM.docx]

**Additional file 1**

Supplementary Table 1. The variables in the 49-item frailty index and their respective scoring criteria;

Supplementary Table 2: Healthy Eating Index -2015 components and scoring standards.

Supplementary Table 1. The variables in the 49-item frailty index and their respective scoring criteria[1]

| Variable |  | Scoring |
| --- | --- | --- |
| Cognition |  |  |
| 1. Experience confusion/memory problems |  | Yes = 1, No= 0 |
| Dependence |  |  |
| 2. Managing money |  | Difficulty = 1, No Difficulty = 0 |
| 3. Stooping, crouching, kneeling |  | Difficulty = 1, No Difficulty = 0 |
| 4. Lifting or carrying |  | Difficulty = 1, No Difficulty = 0 |
| 5. House chore Difficulty |  | Difficulty = 1, No Difficulty = 0 |
| 6. Preparing meals |  | Difficulty = 1, No Difficulty = 0 |
| 7. Standing up from armless chair |  | Difficulty = 1, No Difficulty = 0 |
| 8. Getting in and out of bed difficulty |  | Difficulty = 1, No Difficulty = 0 |
| 9. Using fork, knife, drinking from cup |  | Difficulty = 1, No Difficulty = 0 |
| 10. Dressing yourself |  | Difficulty = 1, No Difficulty = 0 |
| 11. Standing for long periods difficulty |  | Difficulty = 1, No Difficulty = 0 |
| 12. Grasp/holding small objects |  | Difficulty = 1, No Difficulty = 0 |
| 13. Attending social event |  | Difficulty = 1, No Difficulty = 0 |
| 14. Push or pull large objects |  | Difficulty = 1, No Difficulty = 0 |
| 15. Walking for a quarter mile difficulty |  | Difficulty = 1, No Difficulty = 0 |
| 16. Walking up 10 steps difficulty |  | Difficulty = 1, No Difficulty = 0 |
| Depressive Symptoms |  |  |
| 17. Have little interest in doing things |  | Nearly every day = 1, More than half the days = 0.66, Several days = 0.33, Not at all= 0 |
| 18. Feeling down, depressed, or hopeless |  | Nearly every day = 1, More than half the days = 0.66, Several days = 0.33, Not at all= 0 |
| 19. Trouble sleeping or sleeping too much |  | Nearly every day = 1, More than half the days = 0.66, Several days = 0.33, Not at all= 0 |
| 20. Feeling tired or having little energy |  | Nearly every day = 1, More than half the days = 0.66, Several days = 0.33, Not at all= 0 |
| 21. Poor appetite or overeating |  | Nearly every day = 1, More than half the days = 0.66, Several days = 0.33, Not at all= 0 |
| 22. Feeling bad about yourself |  | Nearly every day = 1, More than half the days = 0.66, Several days = 0.33, Not at all= 0 |
| 23. Trouble concentrating on things |  | Nearly every day = 1, More than half the days = 0.66, Several days = 0.33, Not at all= 0 |
| Comorbidities |  |  |
| 24. Arthritis |  | Yes = 1, Suspect = 0.5 No = 0 |
| 25. Thyroid problems |  | Yes = 1, Suspect = 0.5 No = 0 |
| 26. Chronic bronchitis |  | Yes = 1, Suspect = 0.5 No = 0 |
| 27. Cancer |  | Yes = 1, Suspect = 0.5 No = 0 |
| 28. Congestive heart failure |  | Yes = 1, Suspect = 0.5 No = 0 |
| 29. Coronary heart disease |  | Yes = 1, Suspect = 0.5 No = 0 |
| 30. Angina |  | Yes = 1, Suspect = 0.5 No = 0 |
| 31. Heart attack |  | Yes = 1, Suspect = 0.5 No = 0 |
| 32. Stroke |  | Yes = 1, Suspect = 0.5 No = 0 |
| 33. Blood pressure |  | Yes = 1, Suspect = 0.5 No = 0 |
| 34. Diabetes |  | Yes = 1, Suspect = 0.5 No = 0 |
| 35. weak/failing kidneys |  | Yes = 1, Suspect = 0.5 No = 0 |
| 36. Urinary Leakage |  | Yes = 1, Suspect = 0.5 No = 0 |
| Hospital Utilization and Access to Care |  |  |
| 37. Self-rated health |  | Fair, poor = 1, Excellent, Very good, good = 0 |
| 38. Health now compared with 1 year ago |  | Worse =1, About the same, better =0 |
| 39. Overnight hospital patient in past year |  | Yes =1, No =0 |
| 40. Frequency of health care use during past year | | None =0, 1-5 =0,5, More than 5 =1 |
| 41. Number of prescribed medications |  | None =0, 1-4 =0.5, 5 and more =1 |
| Physical Performance and Anthropometry |  |  |
| 42. Body mass index |  | <18.5, ≥30 = 1; 25-<30 = 0.5；18.5-25 = 0 |
| 43. Handgrip strength |  | MALE: For BMI ≤24, GS ≤29; For BMI 24.1-28, GS ≤ 30; For BMI >28, GS ≤ 32 = 1 |
|  |  | FEMALE: For BMI ≤ 23, GS ≤ 17; For BMI 23.1-26, GS ≤ 17.3; For BMI 26.1-29, GS ≤ 18;  For BMI>29, GS ≤ 21 = 1 |
| Laboratory Values |  |  |
| 44. Glycohemoglobin (%) |  | 0%-5.7% =0, >5.7% =1 |
| 45. Red blood cell count (million cells/mL) |  | MALE: 4.7-6.1 =0, Other =1; FEMALEALE: 4.2-5.4 =0, Other =1 |
| 46. Hemoglobin (g/dL) |  | MALE: 13.5-18 =0, Other =1; FEMALEALE: 12-16 =0, Other =1 |
| 47. Red cell distribution width (%) |  | 11.6-14.6 =0, Other =1 |
| 48. Lymphocyte percent (%) |  | 20-40 =0, Other =1 |
| 49. Segmented neutrophils percent (%) |  | 40-80 =0, Other =1 |

Supplementary Table 2. Healthy Eating Index -2015 components and scoring standards[2]

| Component | Maximum points | Standard for maximum score | Standard for minimum score of zero |
| --- | --- | --- | --- |
| Adequacy |  |  |  |
| Total fruits^2^ | 5 | ≥ 0.8 cup equivalents per 1,000 kcal | No fruits |
| Whole fruits^3^ | 5 | ≥ 0.4 cup equivalents per 1,000 kcal | No whole fruits |
| Total vegetables^4^ | 5 | ≥ 1.1 cup equivalents per 1,000 kcal | No vegetables |
| Greens and beans^4^ | 5 | ≥ 0.2 cup equivalents per 1,000 kcal | No dark green vegetables or legumes |
| Whole grains | 10 | ≥ 1.5 oz equivalents per 1,000 kcal | No whole grains |
| Dairy^5^ | 10 | ≥ 1.3 cup equivalents per 1,000 kcal | No dairy |
| Total protein foods^6^ | 5 | ≥ 2.5 oz equivalents per 1,000 kcal | No protein foods |
| Seafood and plant proteins^6,7^ | 5 | ≥ 0.8 oz equivalents per 1,000 kcal | No seafood or plant proteins |
| Fatty acids^8^ | 10 | (PUFAs + MUFAs)/SFAs ≥ 2.5 | (PUFAs + MUFAs)/SFAs ≤ 1.2 |
| Moderation |  |  |  |
| Refined grains | 10 | ≤ 1.8 oz equivalents per 1,000 kcal | ≥ 4.3 oz equivalents per 1,000 kcal |
| Sodium | 10 | ≤ 1.1 gram per 1,000 kcal | ≥ 2.0 grams per 1,000 kcal |
| Added sugars | 10 | ≤ 6.5% of energy | ≥ 26% of energy |
| Saturated fats | 10 | ≤ 8% of energy | ≥ 16% of energy |

Note:

^1^Intakes between the minimum and maximum standards are scored proportionately. The total healthy eating index score is the sum of the adequacy components (i.e. foods to eat more of for good health) and moderation components (i.e. foods to limit for good health).

^2^Includes 100% fruit juice.

^3^Includes all forms except juice.

^4^Includes legumes (beans and peas).

^5^Includes all milk products, such as fluid milk, yogurt, and cheese, and fortified soy beverages.

^6^Includes legumes (beans and peas).

^7^Includes seafood, nuts, seeds, soy products (other than beverages), and legumes (beans and peas).

^8^Ratio of poly- and monounsaturated fatty acids (PUFAs and MUFAs) to saturated fatty acids (SFAs).

Reference:

1. Hakeem FF, Bernabé E, Sabbah W. Association Between Oral Health and Frailty Among American Older Adults. Journal of the American Medical Directors Association. 2021;22:559-563.e2.

2. Krebs-Smith SM, Pannucci TE, Subar AF, Kirkpatrick SI, Lerman JL, Tooze JA, et al. Update of the Healthy Eating Index: HEI-2015. Journal of the Academy of Nutrition and Dietetics. 2018;118:1591–602.
